# Novel heterozygous mutation in MYH3 causes contractures, pterygia, and spondylocarpostarsal fusion syndrome 1: A case report

**DOI:** 10.1097/MD.0000000000043884

**Published:** 2025-08-08

**Authors:** Xuefen Zhu, Qiang Yao

**Affiliations:** a Department of Neonatology, Hangzhou Women’s Hospital, Hangzhou, Zhejiang Province, China.

**Keywords:** contractures, pterygia, and spondylocarpostarsal fusion syndrome, distal arthrogryposis syndrome, MYH3 gene, whole genome sequencing

## Abstract

**Rationale:**

Contractures, pterygia, and spondylocarpotarsal fusion syndrome (CPSFS) comprises a group of extremely rare genetic disorders characterized by congenital craniofacial and musculoskeletal abnormalities. With fewer than 500 cases reported globally, this scarcity contributes to limited clinical recognition, frequent diagnostic delays or errors, and missed opportunities for timely intervention. We present this case to enhance awareness of CPSFS and report a novel pathogenic variant in MYH3 (previously undocumented in the literature) that broadens the known mutational spectrum of MYH3 and enriches the phenotypic profile of CPSFS.

**Patient concerns:**

A female neonate was born at 29 weeks, prenatal ultrasound and magnetic resonance imaging revealed scoliosis and vertebral fusion. The postnatal examination showed microstomia, low-set ears, a short neck with webbing, and flexion contractures at shoulders, elbows, knees, and hands. The whole genome sequencing found novel variants, namely NM_002470.4: c.1914del C; p. Lys639Argfs*18 and NM_002470.4: c.-68 + 4A > T, in the MYH3.

**Diagnoses:**

CPSFS 1.

**Interventions:**

Immediately after birth, noninvasive ventilatory support was initiated. The surgical team conducted comprehensive evaluations, while concurrent genetic testing was performed. Given the infant’s multiple systemic skeletal malformations and inability to sustain spontaneous respiration, surgical intervention was deemed nonviable.

**Outcomes:**

Due to severe thoracic deformity and bronchopulmonary dysplasia, the infant required continuous noninvasive ventilation from birth and remained ventilator-dependent. At a corrected gestational age of 36 weeks and 4 days, life-sustaining therapy was withdrawn following thorough counseling and parental deliberation. The infant died shortly thereafter.

**Lessons:**

Prenatal ultrasound and fetal magnetic resonance imaging can reliably detect characteristic manifestations including scoliosis, joint developmental abnormalities, and clubfoot. Thus, regular prenatal surveillance plays a critical role in early disease identification. For suspected cases, genetic counseling and diagnostic testing enable informed parental decision-making regarding management of affected offspring and future reproductive planning.

## 1. Introduction

Distal arthrogryposis syndrome (DAS) represents a spectrum of inherited bone and joint disorders characterized by significant clinical heterogeneity. The pathogenesis of DAS involves intricate interplay between genetic and environmental factors. Advances in genetic testing have identified mutations in genes such as MYH3, TPM2, TNNI2, ECEL1, TNNT3, and PIEZO2 in DAS development, with MYH3 emerging as the primary causative gene.^[1]^ Contractures, pterygia, and spondylocarpotarsal fusion syndrome 1 (CPSFS 1), a subtype of DAS, is predominantly caused by heterozygous mutations in the MYH3 gene, including single or double allelic variants.^[2]^

In this case report, we describe a severe neonatal presentation of CPSFS 1, where whole genome sequencing identified a heterozygous MYH3 variant.

## 2. Case presentation

A female neonate, the smaller of dichorionic diamniotic twins, was born vaginally at 29 weeks’ gestation to gravida 1, para 2 mother. Birth weight was 1040 g, length 34 cm, and head circumference 25 cm. Apgar scores of 9 both 1 and 5 minutes. Amniotic fluid was clear, with normal umbilical cord and placenta. The parents and the neonate’ older sister are healthy, with no consanguinity or familial history of genetic disorders.

**Figure 1.**
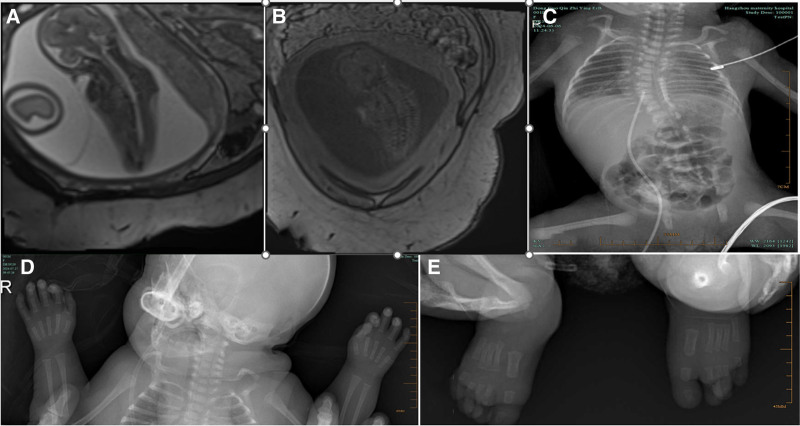
(A and B) MRI revealed spinal scoliosis, thoracolumbar kyphosis, hypoplastic thoracic vertebrae, and vertebral fusion. (C) Chest radiography confirmed scoliosis. (D and E) Wrist and ankle radiographs showed distal flexion contractures of the fingers. No evidence of carpal or tarsal bone fusion was observed. MRI = magnetic resonance imaging.

**Figure 2.**
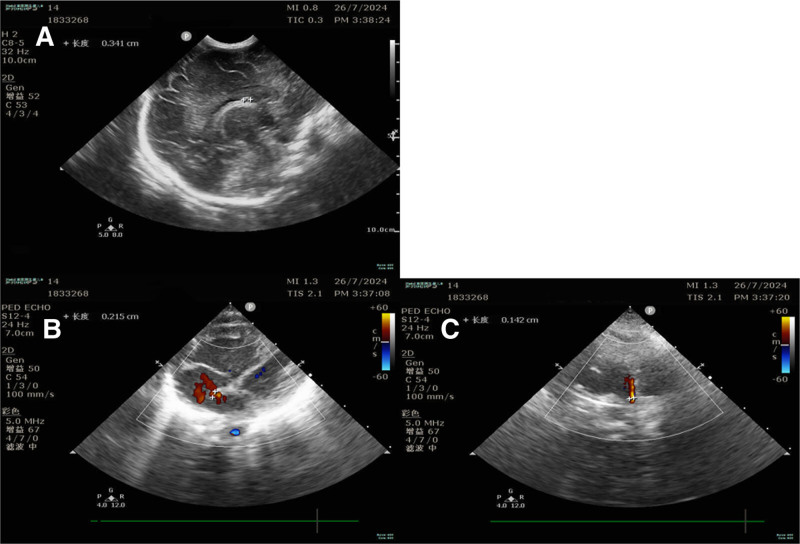
(A) Cranial ultrasound examination revealed subependymal cysts. (B and C) Echocardiography showed a patent foramen ovale (PFO) with a diameter of 0.21 cm. and a patent ductus arteriosus (PDA) with a diameter of 0.14 cm (C).

Routine prenatal ultrasound at 24+ weeks revealed fetal spinal abnormalities. Subsequent fetal magnetic resonance imaging at our institution demonstrated spinal scoliosis, thoracolumbar kyphosis, hypoplastic thoracic vertebrae, and vertebral fusion (Fig. 1A and B). Postnatal transfer to the neonatal intensive care unit occurred due to prematurity and respiratory distress. Physical examination identified microstomia, low-set ears, a short webbed neck, and multiple flexion contractures with limited range of motion at the shoulders, elbows, knees, and distal interphalangeal joints (Fig. 1D and E). Chest radiography confirmed scoliosis (Fig. 1C). Wrist and ankle radiographs showed finger flexion contractures without carpal/tarsal fusion (Fig. 1D and E). Cranial ultrasound revealed subependymal cysts (Fig. 2A), and echocardiography demonstrated a 0.21 cm patent foramen ovale (Fig. 2B) and a 0.14 cm patent ductus arteriosus (Fig. 2C).

Whole genome chromosomal microarray was normal. Whole genome sequencing identified 2 novel heterozygous MYH3 variants: NM_002470.4: c.1914del C; p. Lys639Argfs*18 in exon 17 and NM_002470.4: c.-68 + 4A>T in intron 1. Neither variant is documented in gnomAD database (PM2_P). The frameshift variant (c.1914del C) was classified as a variant of uncertain significance (VUS) per ACMG guidelines (PM2_P). The intronic variant (c.-68 + 4A>T) was predicted by bioinformatics tools to cause abnormal splicing (PP3) and was also classified as VUS (PM2_P+PP3).

Based on clinical and genetic findings, CPSFS 1 was diagnosed. Parental consent for further treatment was withdrawn due to severe thoracic deformity, precluding Sanger sequencing for allelic configuration.

## 3. Discussion

MYH3 mutations underlie several DAS subtypes. DA2A (Freeman–Sheldon syndrome) and DA2B3 (Sheldon–Hall syndrome) predominantly present with craniofacial abnormalities and distal joint contractures. DA2A is characterized by pursing of lips (whistling face) and H- or V-shaped mandibular clefts,^[3]^ whereas DA2B3 features a triangular facial contour and severe microstomia. Both subtypes are associated with congenital camptodactyly.^[4]^ In contrast, CPSFS 1A and CPSFS 1B primarily involve skeletal defects such as scoliosis, vertebral fusion, and pterygia, with occasional mild craniofacial features. Despite the striking similarity in their clinical manifestations, the genetic inheritance patterns of CPSFS 1A and CPSFS 1B differ: the former is autosomal dominant, whereas the latter is autosomal recessive.^[1,5]^ Table 1 summarizes these disorders.

**Table 1:** Differential phenotypes of MYH3-related syndromes.

Phenotype	Contractures, pterygia, and spondylocarpotarsal fusion syndrome 1A (CPSFS 1A)	Contractures, pterygia, and spondylocarpotarsal fusion syndrome 1B (CPSFS 1B)	Freeman–Sheldon syndrome (DA2A)	Sheldon–Hall syndrome (DA2B3)
Inheritance	AD	AR	AD	AD
Face	Low-set posteriorly rotated ears, ptosis, downslanting palpebral fissures, cleft palate	Cleft palate	Whistling face, small mouth, high palate, broad nasal bridge, alar hypoplasia	Triangular face, ptosis, downslanting palpebral fissure
Neck	Short neck	Short neck, webbed neck	Short neck	No data
Spine	Scoliosis, hemivertebra, vertebral fusion	Scoliosis, cervical fusion, thoracic fusion	Scoliosis, spina bifida occulta	Scoliosis
Hands and feet	Congenital multiple joint contractures (elbow, hip, knee), congenital finger flexion, carpal fusion tarsal fusion	Tarsal fusion, clubfoot	Congenital multiple joint contractures (shoulder, elbow, wrist, hip, knee, interphalangeal), clubfoot, finger flexion	Clubfoot, congenital finger flexion

AD = autosomal dominant, AR = autosomal recessive.

Our patient presents a distinct facial appearance characterized by a small mouth, low-set ears, pterygia distributed throughout the body, including the neck, axillae, elbows, and knees, scoliosis, and vertebral fusion, fulfilling the diagnostic criteria for CPSFS 1. Rare phenotypes included cardiac defects (patent foramen ovale, patent ductus arteriosus) and subependymal cysts. Yang et al^[6]^ reported similar neural anomalies (temporal arachnoid cyst, occipital edema), suggesting MYH3 mutations may disrupt neurodevelopment.

MYH3 (chromosome 17p13.1) encodes myosin heavy chain 3. Its expression exhibits temporal specificity, as it is transcribed and translated into MYH3 protein exclusively in embryonic muscle cells and osteoblasts. It plays a pivotal role in the normal development of fetal vertebral bodies and the modulation of muscle contraction and relaxation.^[1]^ Following birth, the expression of MYH3 protein diminishes rapidly and is only reactivated during skeletal muscle regeneration subsequent to skeletal muscle injury in adults.^[7]^

Recent studies indicate a potential correlation between MYH3 mutation sites and genotype–phenotype. Pathogenic variants in head domain predominantly manifest as craniofacial abnormalities and distal arthrogryposis, with skeletal defects occurring infrequently. In contrast, tail domain variants primarily exhibit severe skeletal malformations, occasionally presenting concurrent distinctive craniofacial features.^[4,8]^ Previous literature indicates that pathogenic variants associated with DA2A and DA2B3 predominantly localize to the head domain, whereas variants linked to CPSFS 1 occur in both head and tail domain.^[6,9]^ In the present case, the patient’s mutation site is situated in the head domain of the MYH3 protein. This observation demonstrates symptom overlaps across these disorders while revealing significant correlations between specific mutation loci and distinct phenotypic spectra.

Currently, no curative treatment for CPSFS 1 exists, the management of CPSFS 1 necessitates a multidisciplinary approach due to its complex multisystem involvement. Consequently, early diagnosis and intervention are critical for optimizing long-term functional outcomes. Prenatal screening and genetic testing are critical for early diagnosis. Ultrasound examination during pregnancy, particularly between the 20th and 25th weeks, can be utilized for screening purposes.^[10]^ In cases where suspicion arises, in conjunction with genetic testing. In the present case, fetal spinal abnormalities were identified during an ultrasound examination at 24+ weeks of gestation, and subsequent postnatal genetic testing confirmed the MYH3 mutation.

This case report details the diagnosis and management of CPSFS 1 in a single patient, with inherent limitations in generalizability. Furthermore, institutional disparities in diagnostic equipment, expertise, and experience pose additional barriers to consistent case recognition and management.

## 4. Conclusion

Owing to the rarity of CPSFS 1 and phenotypic overlap among its subtypes, diagnosis and treatment pose considerable challenges. Thus, enhancing clinical awareness is imperative. Key recommendations include: ultrasonographers recognizing characteristic prenatal sonographic features to enable early detection. Neonatologists maintaining proficiency in neonatal diagnosis and management, particularly respiratory support and multisystem assessment. Surgeons determining optimal timing for interventions to maximize functional recovery. Implementing multidisciplinary protocols through such targeted collaboration significantly improves functional outcomes and quality of life.

## Author contributions

**Data curation:** Qiang Yao.

**Formal analysis:** Xuefen Zhu, Qiang Yao.

**Funding acquisition:** Xuefen Zhu, Qiang Yao.

**Investigation:** Qiang Yao.

**Resources:** Xuefen Zhu.

**Writing – original draft:** Xuefen Zhu, Qiang Yao.

**Writing – review & editing:** Xuefen Zhu, Qiang Yao.
